# Clinical *Mycobacterium tuberculosis* isolates exhibit a molecular clock rate below 1 SNP per genome per year

**DOI:** 10.3389/fmicb.2025.1591792

**Published:** 2025-06-13

**Authors:** Jun-Li Wang, Ya-Li Chen, Cui-Ping Guan, Kan Yu, Mao-Shui Wang

**Affiliations:** ^1^Department of Lab Medicine, The Affiliated Hospital of Youjiang Medical University for Nationalities, Baise, China; ^2^Guangxi Engineering Research Center for Precise Genetic Testing of Long-Dwelling Nationalities, Guangxi, China; ^3^Engineering Research Center of Guangxi Higher Education Institutions for Precise Genetic Testing of Long-Dwelling Nationalities, Guangxi, China; ^4^Department of Lab Medicine, Shandong Public Health Clinical Center, Shandong University, Jinan, China; ^5^Marshall Centre, Division of Infection and Immunity, School of Biomedical Sciences, University of Western Australia, Perth, WA, Australia; ^6^The Marshall Centre for Infectious Diseases, Research and Training, The University of Western Australia, Perth, WA, Australia; ^7^School of Biomedical Sciences, The University Western Australia, Perth, WA, Australia

**Keywords:** molecular clock, *Mycobacterium tuberculosis*, single nucleotide polymorphism, heterogeneity, systematic review

## Abstract

**Purpose:**

Tuberculosis (TB) remains a significant global health concern, necessitating effective measures to control the epidemic. Understanding the evolution of *Mycobacterium tuberculosis* (*M. tb*) through molecular clock analysis is crucial for tracing outbreaks, managing transmission, and ultimately improving TB management in practice.

**Results:**

A total of 27 studies were included for analysis. The pooled mutation rate was estimated at 0.63 single nucleotide polymorphisms (SNPs) per genome per year [95% confidence interval (CI): 0.51–0.75; 95% predictive interval (PI): 0.04–1.22], significant heterogeneity (I^2^ = 92.7%, *p* < 0.001) was observed. Clinical strains had a mutation rate of 0.55 SNPs per year (95% CI: 0.45–0.65; 95% PI: 0.12–0.98), while model strains showed a higher rate of 1.14 SNPs per year (95% CI: 0.68–1.60; 95% PI: −0.22-2.50; Meta-regression analysis, *p* = 0.006). Mutation rates did not significantly differ between transmission events and reactivation or single episodes (*p* = 0.497).

**Conclusion:**

The mutation rate of clinical *M. tb* strains is below 1 SNP per genome per year, indicating evolutionary stability in clinical settings. This finding is important for TB outbreak reconstructions and public health strategies. Future research should refine subgroup analyses based on infection characteristics for more precise molecular clock estimates.

**Systematic review registration:**

PROSPERO, identifier CRD42024595161.

## Introduction

Tuberculosis (TB) remains a major global health concern, with 10.6 million new cases and 1.3 million deaths reported by the WHO in 2022. Molecular clock analysis, which could examine the genetic evolution of *Mycobacterium tuberculosis* (*M. tb*), is critical for understanding TB outbreaks and the emergence of drug-resistant strains. This method estimates the timing of evolutionary events by assuming mutations accumulate at a relatively constant rate. However, determining *M. tb*’s precise molecular clock rate remains a challenge, with estimates ranging from 0.13 to over 6 single nucleotide polymorphisms (SNPs) per genome per year, varying across epidemiological contexts (Colangeli et al., 2014; Copin et al., 2016). Factors such as lineage type, immune pressure, and antibiotic exposure may influence its variability. Embracing this variability through a meta-analysis of clinical data can improve both replicability and generalizability in biomedical research (Usui et al., 2021). In this context, this systematic review aims to gather and synthesize existing evidence to generate a pooled estimate of the molecular clock rate for *M. tb*, providing more reliable insights into its evolutionary dynamics. Such consistent estimate will aid in constructing accurate epidemiological links and support more effective interventions during TB outbreaks.

## Methods

This systematic review and meta-analysis followed a registered protocol in PROSPERO (CRD42024595161) and adhered to PRISMA guidelines to ensure thorough methodology (Page et al., 2021). The aim was to estimate mutation rates in *M. tb* and assess contributing factors to its variability.

A comprehensive literature search of PubMed, Scopus, Web of Science, and Embase was conducted as of August 16, 2024, using keywords related to, but not limited to: *M. tb* and mutation rates (Supplementary material, p. 1). Literature selection, data extraction, and quality assessment were independently conducted by two authors (CYL and GCP), with a third author (WJL) consulted for disagreements. Studies were eligible if they (1) directly reported mutation rates, and (2) included mutants or SNPs identified during a specified period. Exclusion criteria included reviews, conference meeting, editorials, or studies lacking sufficient quantitative data; non-English literature; duplicate studies; replicated datasets, and those relying solely on database-derived information or involving drug-resistance mutations. Additionally, Indel and other structural variants were excluded and not included for analysis.

The quality of included studies was assessed using the AXIS tool, considering elements such as sample size justification, methodology, and result reporting (Downes et al., 2016). No studies were excluded based on quality, as there are no established quantitative thresholds for such exclusions.

Pooled mutation rates were estimated using a random-effects model with 95% confidence intervals (CI) and predictive intervals (PI), employing the metan command in Stata/SE (v18.0). Heterogeneity was quantified using the I^2^ statistic and the Chi-square test, with I^2^ > 75% or *p* < 0.05 indicating significant heterogeneity. Subgroup analyses assessed variation in mutation rates by model versus clinical strains and transmission versus “reactivation or single infection episode,” and sources of heterogeneity were assessed using Meta-regression analysis. Publication bias was not evaluated, as established methods for assessing bias in single-arm meta-analyses may be unreliable (Hunter et al., 2014).

## Results

### Literature selection

**Figure 1:**
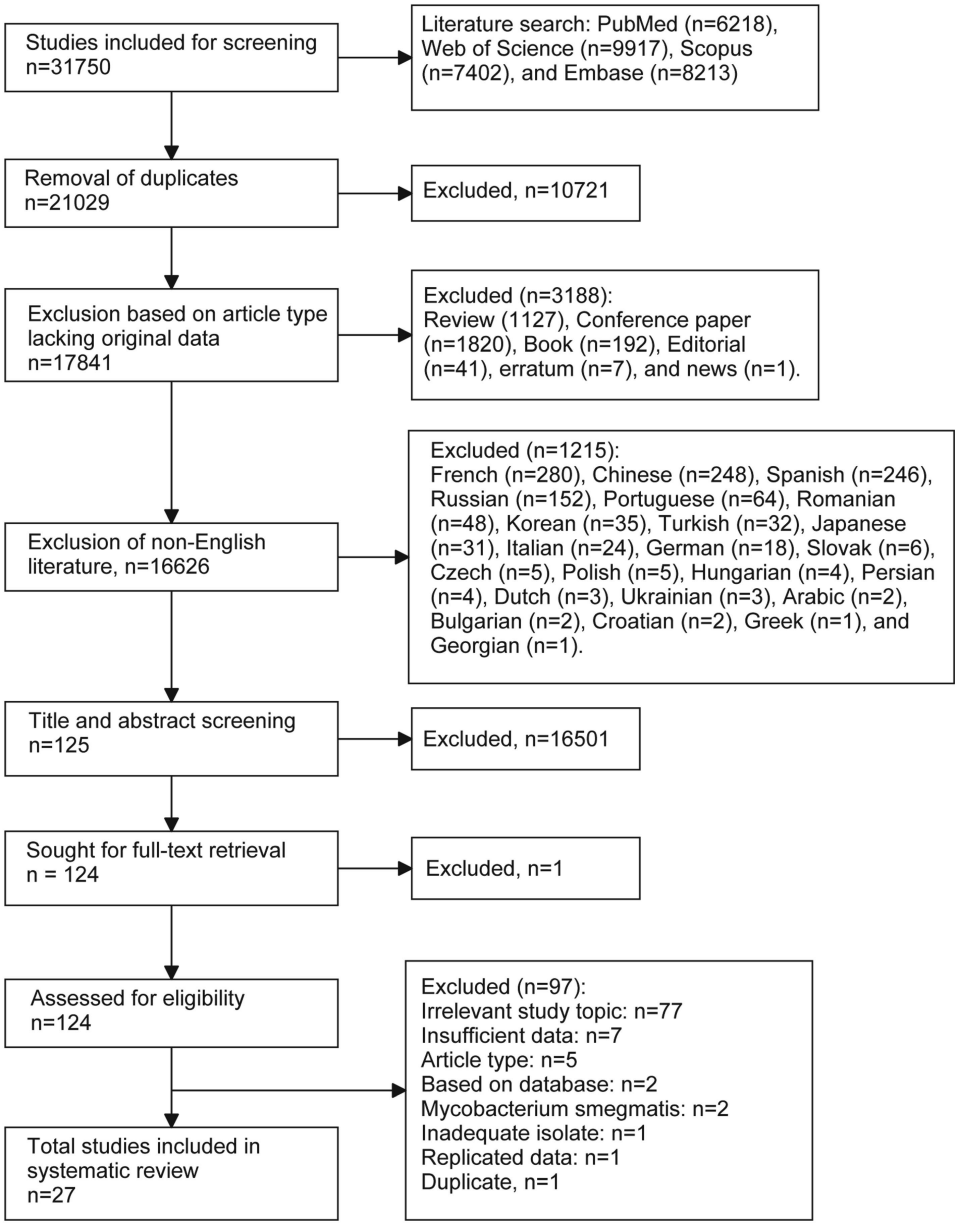
Flow chart of the literature selection process.

Overall, 31,750 citations were identified through database searches. After removing duplicates (*n* = 10,721) and other unsuitable articles (*n* = 3,188), including inappropriate document types (*n* = 3,188) and non-English publications (*n* = 1,215), 16,626 records were screened by title and abstract for eligibility. Of these, 16,501 were excluded, leaving 125 citations identified as relevant or potentially relevant for full-text screening. Following further exclusions (*n* = 97), 27 studies were included in the final analysis (Figure 1).

### Basical characteristics

Table 1 provides a comprehensive summary of the characteristics of the studies included in this meta-analysis. Of these included studies, three were bench studies (Copin et al., 2016; Ford et al., 2011; Comas et al., 2011) and other were cohort. Most cohort studies (23 out of 24) were conducted retrospectively, with only one study being prospective (Roetzer et al., 2013). One study utilized culture media (Comas et al., 2011), while two were conducted on animal models: one on Cynomolgus macaques (Ford et al., 2011) and the other on mice (Copin et al., 2016). In three studies, model isolates [Erdman (Ford et al., 2011), CDC1551 (Comas et al., 2011), T85 (Comas et al., 2011), and H37Rv (Copin et al., 2016)] were measured, whereas clinical isolates were evaluated in the remaining 24 studies. In eight studies, isolates were collected from within a single host (either reactivation or a single infection episode). The remaining 18 studies involved isolates collected from transmission events between patients. Additionally, six studies (with eight datasets) reported single or dual mutation rates without accompanying 95% CI; these rates were combined to calculate the pooled mean and 95% CI. Three studies (with seven datasets) reported means and 95% highest posterior density (HPD), and the 95% HPD was used in place of the 95% CI for the pooled estimate (Bainomugisa et al., 2021; Kuhnert et al., 2018; Merker et al., 2018). The remaining 19 studies (with 35 datasets) provided both the mean mutation rates and their corresponding 95% CI.

**Table 1:** Characteristics of included studies (*n* = 27) for estimating the mutation rate of *Mycobacterium tuberculosis* isolates.

Sequence	Study characteritics				*M. tuberculosis*				Mutation rate (SNPs/genome/year)
	First author, year	Country	Study period	Study design	Host	Isolates	Transmission	Primary data or Calculation (original unit)	
1.1	Ford et al. (2011)	USA	–	Bench studies	Cynomolgus macaques	*M. tuberculosis* (Erdman)	Single episode	Calculation (SNPs/bp/day)^a^	1.28 (1.15, 1.40)
1.2	Ford et al. (2011)	USA	–	Bench studies	Cynomolgus macaques	*M. tuberculosis* (Erdman)	Latent status	Calculation (SNPs/bp/day)^a^	0.73 (0.19, 2.68)
1.3	Ford et al. (2011)	USA	–	Bench studies	Cynomolgus macaques	*M. tuberculosis* (Erdman)	Reactivation	Calculation (SNPs/bp/day)^a^	0.91 (0.41, 2.12)
1.4	Ford et al. (2011)	USA	–	Bench studies	Cynomolgus macaques	*M. tuberculosis* (Erdman)	*In vitro* culture	Calculation (SNPs/bp/day)^a^	0.91 (0.43, 1.42)
2	Sandegren et al. (2011)	Sweden	1996–2010	Retrospective corhort	Human	Clinical isolates	Transmission	Calculation (SNPs and years)	0.44 and 0
3	Saunders et al. (2011)	UK	–	Retrospective corhort	Human	Clinical isolates	Transmission	Calculation (SNPs and years)	2
4	Comas et al. (2011)	USA	–	Bench studies	7H9 media	*M. tuberculosis* (CDC1551; T85)	*In vitro* culture	Calculation (SNPs and years)	5.00 (2.10, 7.91)
5	Bryant et al. (2013)	Netherlands	1992–2007	Retrospective corhort	Human	Clinical isolates	Transmission	Primary data	0.27 (0.13, 0.41)
6	Ford et al. (2013)	Canada	2006	Retrospective corhort	Human	Clinical isolates	Transmission	Calculation (SNPs/bp/day)^a^	0.63 (0.54, 0.72)
7	Kato-Maeda et al. (2013)	USA	1991–2003	Retrospective corhort	Human	Clinical isolates	Transmission	Calculation (SNPs and years)	1.78 (0.11, 3.45)
8	Roetzer et al. (2013)	Germany	1997–2010	Prospective cohort	Human	Clinical isolates	Transmission	Calculation (SNPs/bp/year)^a^	0.44 (0.26, 0.66)
9.1	Walker et al. (2013)	UK	1994–2011	Retrospective corhort	Human	Clinical isolates	Reactivation or Single episode	Primary data	0.3 (0, 0.6)
9.2	Walker et al. (2013)	UK	1994–2011	Retrospective corhort	Human	Clinical isolates	Transmission	Primary data	0.6 (0.3, 0.9)
10.1	Colangeli et al. (2014)	New Zealand	1991–2011	Retrospective corhort	Human	Clinical isolates	Transmission	Calculation (SNPs/bp/generation)^a^	1.95 and 0.64
10.2	Colangeli et al. (2014)	New Zealand	1991–2011	Retrospective corhort	Human	Clinical isolates	Reactivation	Calculation (SNPs/bp/generation)^a^	0.13 and 0.22
11	Eldholm et al. (2014)	Norway	2014	Retrospective corhort	Human	Clinical isolates	Transmission	Primary data	1.1 (0.7, 1.6)
12	Cohen et al. (2015)	South Africa	2008–2013	Retrospective corhort	Human	Clinical isolates	Transmission	Primary data	0.61
13.1	Guerra-Assuncao et al. (2015)	Malawi	1995–2010	Retrospective corhort	Human	Clinical isolates	Transmission	Primary data	0.26 (0.21, 0.31)
13.2	Guerra-Assuncao et al. (2015)	Malawi	1995–2010	Retrospective corhort	Human	Clinical isolates	Reactivation	Primary data	0.45 (0.15, 0.75)
14	Copin et al. (2016)	USA	–	Bench studies	Mouse	*M. tuberculosis* (H37Rv)	Transmission	Primary data	6.43 and 1.28
15	Korhonen et al. (2016)	Finland	1995–2013	Retrospective corhort	Human	Clinical isolates	Transmission	Calculation (SNPs and years)	0.96 (0.24, 1.68)
16	Lillebaek et al. (2016)	Denmark	1960s	Retrospective corhort	Human	Clinical isolates	Transmission	Calculation (SNPs and years)	0.24 and 0.30
17	Folkvardsen et al. (2017)	Denmark	1992–2014	Retrospective corhort	Human	Clinical isolates	Transmission	Primary data	0.24 (0.19, 0.29)
18	Herranz et al. (2017)	Spain and Latvia	2012–2015	Retrospective corhort	Human	Clinical isolates	Reactivation or Single episode	Calculation (SNPs and years)	0.95 (0.56, 1.35)
19.1	Kuhnert et al. (2018)	Switzerland, USA, and Thailand	1987–2012	Retrospective corhort	Human	Clinical isolates	Transmission	Primary data	0.72 (0.40, 1.24)^b^
19.2	Kuhnert et al. (2018)	Switzerland, USA, and Thailand	1987–2012	Retrospective corhort	Human	Clinical isolates	Transmission	Primary data	0.80 (0.41, 1.50)^b^
19.3	Kuhnert et al. (2018)	Switzerland, USA, and Thailand	1987–2012	Retrospective corhort	Human	Clinical isolates	Transmission	Primary data	0.83 (0.42, 1.60)^b^
19.4	Kuhnert et al. (2018)	Switzerland, USA, and Thailand	1987–2012	Retrospective corhort	Human	Clinical isolates	Transmission	Primary data	0.55 (0.32, 0.86)^b^
19.5	Kuhnert et al. (2018)	Switzerland, USA, and Thailand	1987–2012	Retrospective corhort	Human	Clinical isolates	Transmission	Primary data	0.36 (0.11, 0.57)^b^
20	Merker et al. (2018)	Uzbekistan	2001–2006	Retrospective corhort	Human	Clinical isolates	Transmission	Primary data	0.41 (0.32, 0.51)^b^
21	Xu et al. (2018)	China	–	Retrospective corhort	Human	Clinical isolates	Reactivation or Single episode	Primary data	3.2
22.1	Colangeli et al. (2020)	Brazil	2008–2013	Retrospective corhort	Human	Clinical isolates	Reactivation	Primary data	1.80 (0.80, 4.05)
22.2	Colangeli et al. (2020)	Brazil	2008–2013	Retrospective corhort	Human	Clinical isolates	Reactivation	Primary data	0.82 (0.37, 1.84)
23	Godfroid et al. (2020)	Central Asia	1995–2015	Retrospective corhort	Human	Clinical isolates	Transmission	Primary data	0.54 (0.44, 0.63)
24	Bainomugisa et al. (2021)	Malaysia	2012–2017	Retrospective corhort	Human	Clinical isolates	Transmission	Primary data	0.56 (0.23, 0.79)^b^
25.1	Comín et al. (2022)	Spain	2004–2019	Retrospective corhort	Human	Clinical isolates	Reactivation (1–2 years)	Primary data	0.60 (0.26, 1.39)
25.2	Comín et al. (2022)	Spain	2004–2019	Retrospective corhort	Human	Clinical isolates	Reactivation (2–14 years)	Primary data	0.59 (0.31, 1.12)
26.1	Sadovska et al. (2023)	Latvia	2002–2019	Retrospective corhort	Human	Clinical isolates	Single episode	Calculation (SNPs and years)	0.39 (0.01, 0.77)
26.2	Sadovska et al. (2023)	Latvia	2002–2019	Retrospective corhort	Human	Clinical isolates	Reactivation	Calculation (SNPs and years)	0.25 (0.16, 0.34)
27	Zhang et al. (2024)	China	2009–2016	Retrospective corhort	Human	Clinical isolates	Single episode	Primary data	1.20 (1.02, 1.38)

a During the conversion from other units (e.g., SNPs/bp/day), calculations were based on the following parameters: (1) genome size of 4,411,532 bp (source: https://www.ncbi.nlm.nih.gov/nuccore/NC_000962.2); (2) generation time of 18 h; and (3) one year equal to 365 days.

b 95% HPD (Highest Posterior Density) is used instead of the 95% confidence interval.

SNPs, single nucleotide polymorphisms.

### Bias assessment

The bias assessment results, detailed in Supplementary Table 1 (Supplementary materials, p. 2–6), revealed several limitations across the included studies. None of the studies provided justification for their sample sizes. Due to the nature of bench studies and retrospective designs, only a few studies discussed the exclusion process, and no studies implemented measures to address or categorize non-responders. However, two studies (7.4%) provided detailed exclusion results. Eight studies (29.6%) did not report mutation rates directly; these rates were instead calculated based on the number of SNPs and the specified study period. Additionally, 14 studies (51.9%) did not report 95% CI due to either reporting a single rate or lacking sufficient information. Ten studies (37.0%) failed to acknowledge limitations in their findings, and one study (3.7%) did not provide information on funding sources. Furthermore, 13 studies (48.1%) did not obtain ethical approval, as the authors considered their research exempt from this requirement.

### Mutation rate

**Figure 2:**
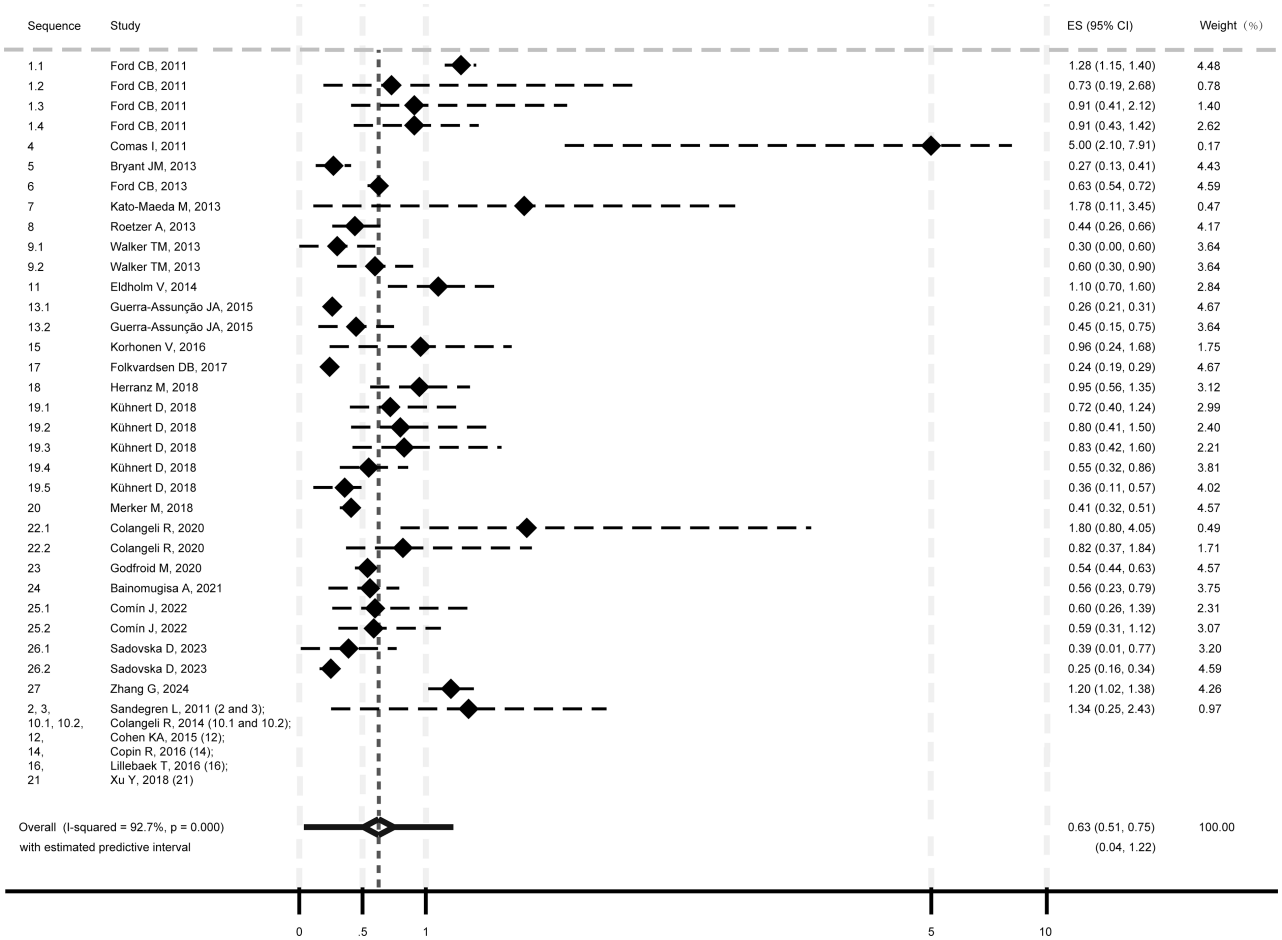
Pooled mutation rate estimate of *Mycobacterium tuberculosis* isolates.

The pooled mutation rate for *M. tb* was estimated at 0.63 SNPs/genome/year (95% CI: 0.51–0.75; I^2^ = 92.7%, *p* < 0.001; Heterogeneity: I^2^ = 92.7%, *p* < 0.001; Figure 2), with a 95% PI of 0.04 to 1.22.

Subgroup analyses showed mutation rates of 1.14 SNPs/genome/year (95% CI: 0.68–1.60; 95% PI: −0.22-2.50; Supplementary Figure 1, p. 7) for model strains (I^2^ = 58.8%, *p* = 0.046) and 0.55 SNPs/genome/year (95% CI: 0.45–0.65; 95% PI: 0.12–0.98) for clinical strains (I^2^ = 88.1%, *p* < 0.001). Transmission isolates had a rate of 0.50 SNPs/genome/year (95% CI: 0.40–0.60; 95% PI: 0.14–0.86; Heterogeneity: I^2^ = 85.9%, *p* < 0.001; Supplementary Figure 2, p. 8), while “reactivation or single episode” isolates showed 0.64 SNPs/genome/year (95% CI: 0.33–0.95; 95% PI: −0.43 to 1.72; Heterogeneity: I^2^ = 90.7%, *p* < 0.001). Meta-regression indicated heterogeneity was significantly associated with isolate type (model vs. clinical; *p* = 0.006), but not transmission/ “reactivation or single episode” (*p* = 0.497).

## Discussion

TB remains a significant global health challenge, with *M. tb* continually evolving to resist existing treatments. Understanding the molecular clock of *M. tb* is crucial for tracking drug resistance, mapping transmission networks, and designing effective interventions. Our systematic review and meta-analysis estimate the mutation rate at 0.63 SNPs/genome/year, with significant variation between model and clinical strains. Regarding clinical *M. tb* strains, the mutation rate is typically below 1 SNP/genome/year, supported by a 95% PI of 0.12–0.98. This estimate aligns well with existing molecular clock data and appears more precise than earlier findings, which ranged from 0.04 to 2.2 SNPs/genome/year (Menardo et al., 2019). Overall, this mutation rate is slower than previously thought, as rates below 10 SNPs were often used as a criterion for recent infection and reactivation (Sadovska et al., 2023). The observed rate underscores the evolutionary stability of *M. tb* in clinical environments, despite host immune pressures and treatment regimens (Nimmo et al., 2020; Reiling et al., 2018). In contrast, model strains showed a higher mutation rate of 1.14 SNPs/genome/year. This discrepancy may be due to fewer environmental constraints in model strains, leading to a higher accumulation of mutations (Perrier, 2020), as well as differences in generation times between model and clinical strains (Colangeli et al., 2014). These findings highlight the need to consider experimental context when interpreting the evolutionary dynamics of *M. tb*.

Furthermore, although isolates derived from transmission events exhibited a lower mutation rate (0.50 SNPs per genome per year) compared to those from reactivation or single infection episodes (0.64 SNPs per genome per year), this difference was not statistically significant. This suggests a potentially rapid mutation rate during reactivation (or single infection episode), likely due to gradual mutation accumulation over time. While transmission imposes selective pressures for host adaptation (Brites and Gagneux, 2012), the overall mutation rate remained slow. This may be explained by that (1) the establishment of infection typically requiring fewer than 10 *M. tb* isolates, thereby limiting genetic diversity during transmission (Treibert et al., 2018); and (2) transmission not relying on rapid evolutionary changes, suggesting that significant mutations may not be immediately necessary.

These findings have important implications for public health and TB control strategies. Establishing that the mutation rate of clinical isolates is typically below 1 SNP per genome per year allows for greater accuracy in outbreak reconstructions and transmission network modeling. The molecular clock offers valuable insights into the genetic evolution of *M. tb*, particularly by helping to identify the timeline of outbreaks, track the spread of resistant strains, and understand transmission dynamics. Most importantly, this precise mutation rate estimate will aid in establishing criteria for distinguishing reactivation from recent infection, accounting for the interval between isolates.

Despite valuable insights, this study has several limitations, including significant heterogeneity, inconsistent study characteristics, reliance on indirect SNP calculations (partly), lack of confidence intervals (partly), and potential bias from pooling single-rate estimates for summary analysis. Another important limitation is the inclusion of datasets derived from diverse geographic and demographic settings. Geographic heterogeneity likely reflects differences in lineage distribution (Brenner and Sreevatsan, 2023), while demographic variability may correspond to the emergence of locally adapted variants (Correa-Macedo et al., 2019). Furthermore, differences in sequencing technologies, bioinformatics pipelines, reference genomes, and variant-calling thresholds across studies can influence the sensitivity and specificity of SNP detection, leading to variability in reported mutation rates. For instance, studies using higher-depth sequencing or more stringent variant-calling criteria may detect fewer mutations, resulting in lower mutation rate estimates, whereas more permissive pipelines could inflate the apparent rate (Koboldt, 2020; Feng et al., 2023). Variations in reference genomes can also introduce systematic biases by affecting alignment accuracy and SNP calling (Valiente-Mullor et al., 2021; Zverinova and Guryev, 2022). Collectively, these methodological discrepancies may contribute to the heterogeneity observed in our meta-analysis and limit the comparability of results across studies. To standardize mutation rates, a fixed genome size corresponding to the H37Rv reference strain (4,411,532 bp) was adopted. Minor discrepancies (<2%) may result from slight variations in genome size across different strains (Sanoussi et al., 2021). Additionally, the limited number of studies involving model isolates constrains the statistical power and generalizability of comparisons with clinical isolates. Therefore, future studies incorporating larger datasets of model isolates are warranted to validate and extend these findings.

In conclusion, this study provides a comprehensive analysis of the molecular clock rates of *M. tb*, highlighting significant variations between model and clinical strains, as well as substantial heterogeneity that underscores the large variation in clock rates across different infection scenarios. Future research should prioritize the collection of more detailed characteristics of each *M. tb* infection event and its context to enhance the precision of molecular clock estimates.

## Data Availability

The original contributions presented in the study are included in the article/Supplementary material, further inquiries can be directed to the corresponding author.
