# Supplementary material for: Clinical Mycobacterium tuberculosis isolates exhibit a molecular clock rate below 1 SNP per genome per year
Source: Front Microbiol. 2025 Jun 13;16:1591792. doi: 10.3389/fmicb.2025.1591792 (PMC12202658; doi:10.3389/fmicb.2025.1591792)
Supplement: Supplementary file 1 [file Data_Sheet_1.pdf]

1. Literature search

1.1 Searching strategies

(evolut* OR phylogen* OR (biological clock*) OR (single nucleotide polymorphism*) OR SNP OR muta* OR ((genom* OR genet*) AND (varia* OR dynam* OR divers*))) AND (frequen* OR rate* OR (per adj5 genome) OR (per adj5 year) OR (per adj5 generation) OR (per adj5 replication) OR (per adj5 base*) OR (per adj5 nucleotide*)) AND (tubercu* OR PTB OR TB)

The searching strategies were transformed between databases using Polyglot (https://polyglot.sr-accelerator.com/).

1.2 Searching time

Aug 16th, 2024

Table 1. Quality assessment results of included studies (n=27) using the AXIS tool.

Included studies		Introduction		Methods									Results					Discussion		Other	
Sequence	First author, year	Q1	Q2	Q3	Q4	Q5	Q6	Q7	Q8	Q9	Q10	Q11	Q12	Q13	Q14	Q15	Q16	Q17	Q18	Q19	Q20
1	Ford CB, 2011 [1]	Yes	Yes	No	NA	NA	NA	NA	Yes	Yes	Yes	Yes	NA	NA	NA	Yes	Yes	Yes	Yes	Yes	Yes
2	Sandegren L, 2011 [2]	Yes	Yes	No	Yes	Yes	Yes	NA	Yes	No	No	Yes	Yes	NA	NA	Yes	Yes	Yes	Yes	Yes	No
3	Saunders NJ, 2011 [3]	Yes	Yes	No	Yes	Yes	Yes	NA	Yes	No	No	Yes	Yes	NA	NA	Yes	Yes	Yes	No	No	No
4	Comas I, 2011 [4]	Yes	Yes	No	NA	NA	NA	NA	Yes	No	No	Yes	NA	NA	NA	Yes	Yes	Yes	Yes	Yes	No
5	Bryant JM, 2013 [5]	Yes	Yes	No	Yes	Yes	Yes	NA	Yes	Yes	Yes	Yes	Yes	NA	NA	Yes	Yes	Yes	Yes	Yes	No
6	Ford CB, 2013 [6]	Yes	Yes	No	Yes	Yes	Yes	NA	Yes	Yes	Yes	Yes	Yes	NA	NA	Yes	Yes	Yes	No	Yes	No
7	Kato-Maeda M, 2013 [7]	Yes	Yes	No	Yes	Yes	Yes	NA	Yes	No	No	Yes	Yes	NA	NA	Yes	Yes	Yes	Yes	Yes	Yes
8	Roetzer A, 2013 [8]	Yes	Yes	No	Yes	Yes	Yes	NA	Yes	Yes	Yes	Yes	Yes	NA	NA	Yes	Yes	Yes	No	Yes	Yes
9	Walker TM, 2013 [9]	Yes	Yes	No	Yes	Yes	Yes	NA	Yes	Yes	Yes	Yes	Yes	NA	NA	Yes	Yes	Yes	Yes	Yes	No
10	Colangeli R, 2014 [10]	Yes	Yes	No	Yes	Yes	Yes	NA	Yes	Yes	No	Yes	Yes	NA	NA	Yes	Yes	Yes	Yes	Yes	No
11	Eldholm V, 2014 [11]	No	Yes	No	Yes	Yes	Yes	NA	Yes	Yes	Yes	Yes	Yes	NA	NA	Yes	Yes	Yes	No	Yes	Yes
12	Cohen KA, 2015 [12]	Yes	Yes	No	Yes	Yes	Yes	NA	Yes	Yes	No	Yes	Yes	NA	NA	Yes	Yes	Yes	Yes	Yes	Yes
13	Guerra-Assunção JA, 2015 [13]	Yes	Yes	No	Yes	Yes	Yes	NA	Yes	Yes	Yes	Yes	Yes	NA	NA	Yes	Yes	Yes	Yes	Yes	Yes
14	Copin R, 2016 [14]	Yes	Yes	No	NA	NA	NA	NA	Yes	Yes	No	Yes	NA	NA	NA	NA	Yes	Yes	No	Yes	Yes

15	Korhonen V, 2016 [15]	Yes	Yes	No	Yes	Yes	Yes	NA	Yes	No	No	Yes	Yes	NA	NA	Yes	Yes	Yes	No	Yes	Yes
16	Lillebaek T, 2016 [16]	Yes	Yes	No	Yes	Yes	Yes	NA	Yes	No	No	Yes	Yes	NA	NA	Yes	Yes	Yes	No	Yes	No
17	Folkvardsen DB, 2017 [17]	Yes	Yes	No	Yes	Yes	Yes	NA	Yes	Yes	Yes	Yes	Yes	NA	NA	Yes	Yes	Yes	Yes	Yes	No
18	Herranz M, 2018 [18]	Yes	Yes	No	Yes	Yes	Yes	NA	Yes	No	No	Yes	Yes	NA	NA	Yes	Yes	Yes	Yes	Yes	No
19	Kühnert D, 2018 [19]	Yes	Yes	No	Yes	Yes	Yes	NA	Yes	Yes	No	Yes	Yes	NA	NA	Yes	Yes	Yes	Yes	Yes	Yes
20	Merker M, 2018 [20]	Yes	Yes	No	Yes	Yes	Yes	NA	Yes	Yes	No	Yes	Yes	NA	NA	Yes	Yes	Yes	No	Yes	No
21	Xu Y, 2018 [21]	Yes	Yes	No	Yes	Yes	Yes	NA	Yes	Yes	No	Yes	Yes	NA	NA	Yes	Yes	Yes	No	Yes	Yes
22	Colangeli R, 2020 [22]	Yes	Yes	No	Yes	Yes	Yes	NA	Yes	Yes	Yes	Yes	Yes	No	Yes	Yes	Yes	Yes	Yes	Yes	Yes
23	Godfroid M, 2020 [23]	Yes	Yes	No	Yes	Yes	Yes	NA	Yes	Yes	Yes	Yes	Yes	NA	NA	Yes	Yes	Yes	No	Yes	No
24	Bainomugisa A, 2021 [24]	Yes	Yes	No	Yes	Yes	Yes	NA	Yes	Yes	Yes	Yes	Yes	NA	NA	Yes	Yes	Yes	Yes	Yes	Yes
25	Comín J, 2022 [25]	Yes	Yes	No	Yes	Yes	Yes	NA	Yes	Yes	Yes	Yes	Yes	NA	NA	Yes	Yes	Yes	Yes	Yes	Yes
26	Sadovska D, 2023 [26]	Yes	Yes	No	Yes	Yes	Yes	NA	Yes	No	No	Yes	Yes	NA	NA	Yes	Yes	Yes	Yes	Yes	Yes
27	Zhang G, 2024 [27]	Yes	Yes	No	Yes	Yes	Yes	NA	Yes	Yes	Yes	Yes	Yes	NA	NA	Yes	Yes	Yes	Yes	Yes	No

Note:

Q1 Were the aims/objectives of the study clear?

Q2 Was the study design appropriate for the stated aim(s)?

Q3 Was the sample size justified?

Q4 Was the target/reference population (or M. tuberculosis isolates) clearly defined? (Is it clear who the research was about?)

Q5 Was the sample frame taken from an appropriate population base so that it closely represented the target/reference population under investigation?

Q6 Was the selection process likely to select subjects/participants (or M. tuberculosis isolates) that were representative of the target/reference population (M. tuberculosis isolates) under investigation?

Q7 Were measures undertaken to address and categorise non-responders?

Q8 Were the risk factor and outcome variables (such as mutation rate) measured appropriate to the aims of the study?

Q9 Were the risk factor and outcome variables (such as mutation rate) measured correctly using instruments/measurements that had been trialled, piloted or published previously?

Q10 Is it clear what was used to determined statistical significance and/or precision estimates? (e.g. p-values, confidence intervals)

Q11 Were the methods (including statistical methods) sufficiently described to enable them to be repeated?

Q12 Were the basic data adequately described?

Q13 Does the response rate raise concerns about non-response bias?

Q14 If appropriate, was information about non-responders described?

Q15 Were the results internally consistent?

Q16 Were the results presented for all the analyses described in the methods?

Q17 Were the authors' discussions and conclusions justified by the results?

Q18 Were the limitations of the study discussed?

Q19 Were there any funding sources or conflicts of interest that may affect the authors’ interpretation of the results?

Q20 Was ethical approval or consent of participants attained?

References

1. Ford CB, Lin PL, Chase MR, et al. Use of whole genome sequencing to estimate the mutation rate of Mycobacterium tuberculosis during latent infection. Nat Genet **2011**; 43(5): 482-6.
2. Sandegren L, Groenheit R, Koivula T, et al. Genomic stability over 9 years of an isoniazid resistant Mycobacterium tuberculosis outbreak strain in Sweden. PLoS One **2011**; 6(1): e16647.
3. Saunders NJ, Trivedi UH, Thomson ML, Doig C, Laurenson IF, Blaxter ML. Deep resequencing of serial sputum isolates of Mycobacterium tuberculosis during therapeutic failure due to poor compliance reveals stepwise mutation of key resistance genes on an otherwise stable genetic background. J Infect **2011**; 62(3): 212-7.
4. Comas I, Borrell S, Roetzer A, et al. Whole-genome sequencing of rifampicin-resistant Mycobacterium tuberculosis strains identifies compensatory mutations in RNA polymerase genes. Nat Genet **2011**; 44(1): 106-10.
5. Bryant JM, Schurch AC, van Deutekom H, et al. Inferring patient to patient transmission of Mycobacterium tuberculosis from whole genome sequencing data. BMC Infect Dis **2013**; 13: 110.
6. Ford CB, Shah RR, Maeda MK, et al. Mycobacterium tuberculosis mutation rate estimates from different lineages predict substantial differences in the emergence of drug-resistant tuberculosis. Nat Genet **2013**; 45(7): 784-90.
7. Kato-Maeda M, Ho C, Passarelli B, et al. Use of whole genome sequencing to determine the microevolution of Mycobacterium tuberculosis during an outbreak. PLoS One **2013**; 8(3): e58235.
8. Roetzer A, Diel R, Kohl TA, et al. Whole genome sequencing versus traditional genotyping for investigation of a Mycobacterium tuberculosis outbreak: a longitudinal molecular epidemiological study. PLoS Med **2013**; 10(2): e1001387.
9. Walker TM, Ip CL, Harrell RH, et al. Whole-genome sequencing to delineate Mycobacterium tuberculosis outbreaks: a retrospective observational study. Lancet Infect Dis **2013**; 13(2): 137-46.
10. Colangeli R, Arcus VL, Cursons RT, et al. Whole genome sequencing of Mycobacterium tuberculosis reveals slow growth and low mutation rates during latent infections in humans. PLoS One **2014**; 9(3): e91024.
11. Eldholm V, Norheim G, von der Lippe B, et al. Evolution of extensively drug-resistant Mycobacterium tuberculosis from a susceptible ancestor in a single patient. Genome Biol **2014**; 15(11): 490.
12. Cohen KA, Abeel T, Manson McGuire A, et al. Evolution of Extensively Drug-Resistant Tuberculosis over Four Decades: Whole Genome Sequencing and Dating Analysis of Mycobacterium tuberculosis Isolates from KwaZulu-Natal. PLoS Med **2015**; 12(9): e1001880.
13. Guerra-Assuncao JA, Crampin AC, Houben RM, et al. Large-scale whole genome sequencing of M. tuberculosis provides insights into transmission in a high prevalence area. Elife **2015**; 4.
14. Copin R, Wang X, Louie E, et al. Within Host Evolution Selects for a Dominant Genotype of Mycobacterium tuberculosis while T Cells Increase Pathogen Genetic Diversity. PLoS Pathog **2016**; 12(12): e1006111.
15. Korhonen V, Smit PW, Haanpera M, et al. Whole genome analysis of Mycobacterium tuberculosis isolates from recurrent episodes of tuberculosis, Finland, 1995-2013. Clin Microbiol Infect **2016**; 22(6): 549-54.
16. Lillebaek T, Norman A, Rasmussen EM, et al. Substantial molecular evolution and mutation rates in prolonged latent Mycobacterium tuberculosis infection in humans. Int J Med Microbiol **2016**; 306(7): 580-5.
17. Folkvardsen DB, Norman A, Andersen AB, Michael Rasmussen E, Jelsbak L, Lillebaek T. Genomic Epidemiology of a Major Mycobacterium tuberculosis Outbreak: Retrospective Cohort Study in a Low-

Incidence Setting Using Sparse Time-Series Sampling. J Infect Dis **2017**; 216(3): 366-74.

18. Herranz M, Pole I, Ozere I, et al. Mycobacterium tuberculosis Acquires Limited Genetic Diversity in Prolonged Infections, Reactivations and Transmissions Involving Multiple Hosts. Front Microbiol **2017**; 8: 2661.
19. Kuhnert D, Coscolla M, Brites D, et al. Tuberculosis outbreak investigation using phylodynamic analysis. Epidemics **2018**; 25: 47-53.
20. Merker M, Barbier M, Cox H, et al. Compensatory evolution drives multidrug-resistant tuberculosis in Central Asia. Elife **2018**; 7.
21. Xu Y, Liu F, Chen S, et al. In vivo evolution of drug-resistant Mycobacterium tuberculosis in patients during long-term treatment. BMC Genomics **2018**; 19(1): 640.
22. Colangeli R, Gupta A, Vinhas SA, et al. Mycobacterium tuberculosis progresses through two phases of latent infection in humans. Nat Commun **2020**; 11(1): 4870.
23. Godfroid M, Dagan T, Merker M, et al. Insertion and deletion evolution reflects antibiotics selection pressure in a Mycobacterium tuberculosis outbreak. PLoS Pathog **2020**; 16(9): e1008357.
24. Bainomugisa A, Meumann EM, Rajahram GS, et al. Genomic epidemiology of tuberculosis in eastern Malaysia: insights for strengthening public health responses. Microb Genom **2021**; 7(5).
25. Comin J, Cebollada A, Aragonese Working Group on Molecular Epidemiology of T, Samper S. Estimation of the mutation rate of Mycobacterium tuberculosis in cases with recurrent tuberculosis using whole genome sequencing. Sci Rep **2022**; 12(1): 16728.
26. Sadovska D, Nodieva A, Pole I, et al. Advantages of analysing both pairwise SNV-distance and differing SNVs between Mycobacterium tuberculosis isolates for recurrent tuberculosis cause determination. Microb Genom **2023**; 9(3).
27. Zhang G, Sun X, Fleming J, et al. Genetic factors associated with acquired phenotypic drug resistance and its compensatory evolution during tuberculosis treatment. Clin Microbiol Infect **2024**; 30(5): 637-45.

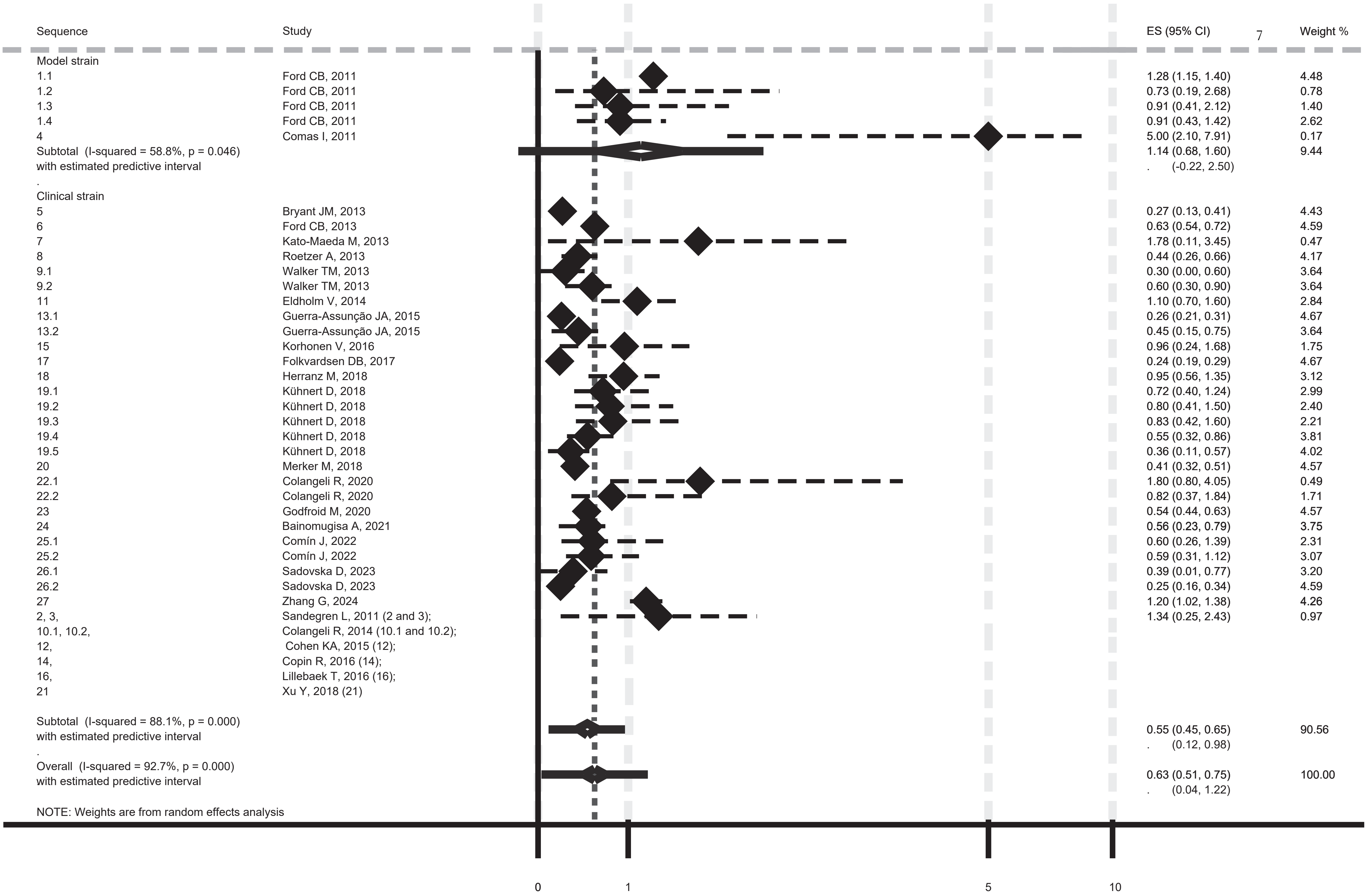

Figure 1. Forest plot: The pooled mutation rate of Mycobacterium tuberculosis (Model vs. Clincal strains).

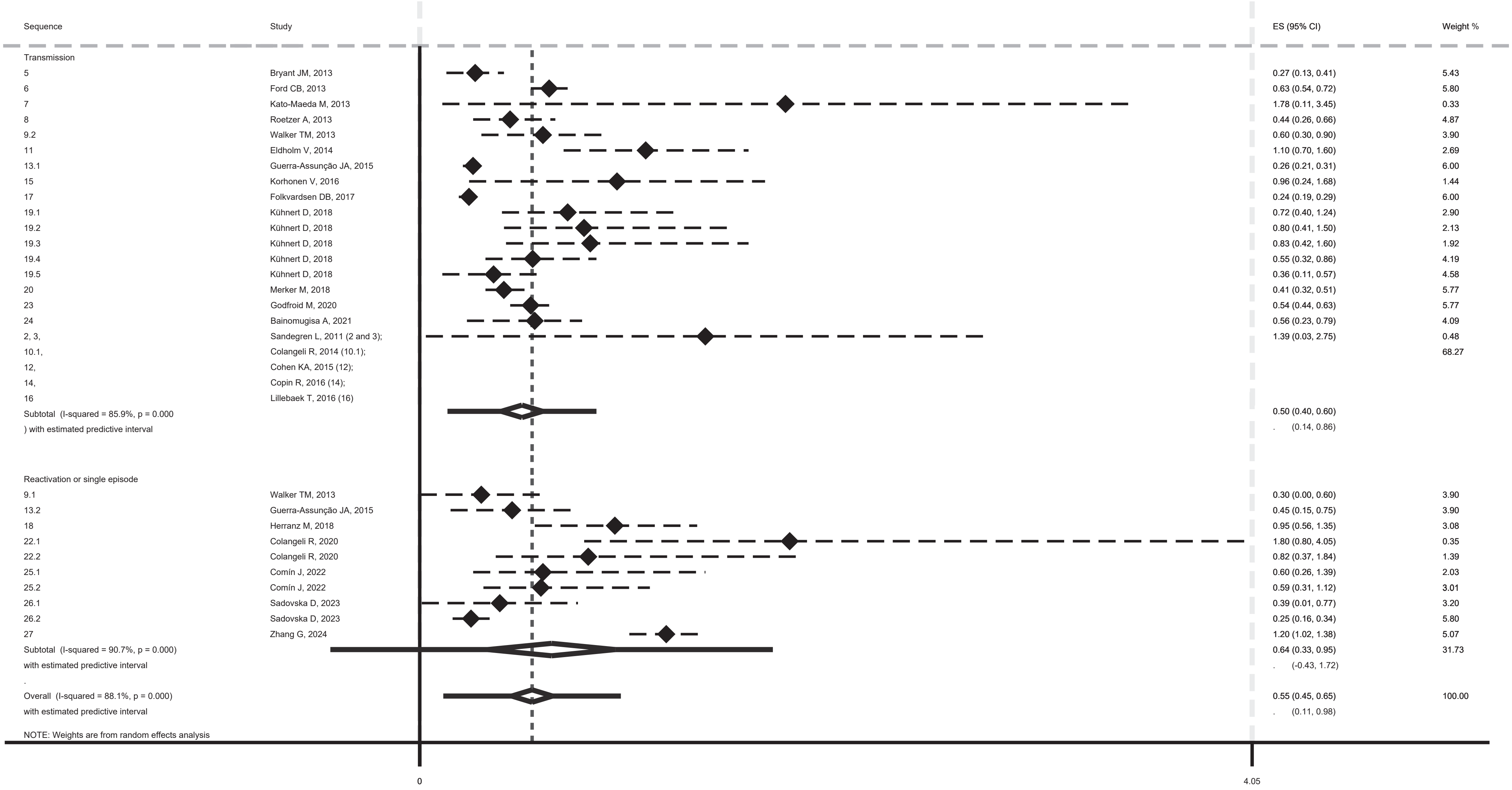

Figure 2. Forest plot: The pooled mutation rate of Mycobacterium tuberculosis (Transmission vs. Reactivation or single episode).

Supplementray information: Genome accession numbers and sequencing details of Mycobacterium tuberculosis isolates from included studies in the systematic review.

Sequence	First author, year	Accession numbers	Sequencing technology	Sequencing coverage	Sequencing depth (fold)	Automation and Pipelines	Reference genome	Thresholds and filters
1	Ford CB, 2011	http://www.broadinstitute.org/annotation/genome/mtb_monkey_reseq.1	Illumina Genome Analyzer	Average: 93%	Average depth: 117	Scaffolded mapping using MAQ v0.7.1<br>De novo assembly using Edena v2.1.1<br>Validation by Sanger sequencing	H37Rv (GenBank accession AL123456); H37Rv (NC_000962)	Reads with average quality score <20 discarded<br>Reads with homopolymeric runs (A/T >9 or G/C >10) discarded<br>Up to 3 mismatches allowed in alignment seed<br>SNP validation through manual inspection and Sanger sequencing
2	Sandegren L, 2011	AEGB00000000, AEGC00000000, and AEGD00000000 (GenBank)	Roche 454-sequencing technology	-	-	CLC Genomics Workbench v3.7	H37Rv (NC_000962); also comparisons with H37Ra (NC_009525), CDC1551 (NC_002755), and F11 (NC_009565)	Average base quality filter cutoff: 15<br>Central base quality filter cutoff: 20<br>Minimum variation frequency cutoff: 75%<br>Maximum ploidy (variation): 2<br>Minimum sequence coverage: 3
3	Saunders NJ, 2011	ERP000450 (EMBL)	Illumina Solexa	-	-	De novo assembly: Velvet<br>Variant calling: MAQ (Mapping and Assembly with Quality)	H37Rv (NC_000962)	Repetitive regions (e.g., transposons, PE/PPE genes) excluded from comparison
4	Comas I, 2011	SRP001097 (NCBI, SRA)	Illumina Genome Analyzer	-	Depth: 302 (range 77–512)	Mapping and SNP calling: MAQ (Mapping and Assembly with Qualities)<br>Annotation: ANNOVAR	H37Rv (NC_000962)	Phred quality score > 30<br>Minimum sequence depth ≥ 5<br>Removed heterozygous calls<br>Excluded mutations in PE/PPE genes and mobile elements
5	Bryant JM, 2013	ERP000111 (EMBL)	Illumina Genome Analyzer GAIIx	Average coverage: 95.6%	Average depth: 100	Read mapping: SMALT<br>Variant calling: samtools and bcftools<br>Additional independent pipeline: RoVar (Robust Variant detection tool)	H37Rv (NC_000962)	≥75% of high-quality mapped reads on each strand supporting the call<br>Base quality score ≥ 50<br>Mapping quality score ≥ 30<br>Reads mapping non-uniquely discarded<br>Variants in repetitive regions (e.g., PE/PPE genes) generally

								excluded
6	Ford CB, 2013	SRA020129 (NCBI, SRA)	Illumina Genome Analyzer	-	-	Scaffolded mapping using MAQ v0.7.1<br>De novo assembly using Edena v2.1.1<br>Validation by Sanger sequencing	H37Rv (NC_000962)	Exclusion of SNPs in repetitive regions (PE_PGRS, PPE genes, transposons)<br>Quality control based on prior experimental validation standards (Sanger resequencing of SNPs)
7	Kato-Maeda M, 2013	No details were available	Illumina Genome Analyzer	Average coverage: 93%	Average depth: 117	Read mapping: BWA (Burrows-Wheeler Aligner)<br>Variant calling: GATK Unified Genotyper<br>Data handling: SAMtools and custom Python scripts	H37Rv (NC_000962)	SNPs confirmed only if ≥85% of reads supported the call and minimum depth ≥12 reads<br>SNPs in repetitive regions (PE, PE_PGRS, PPE families, mobile elements) excluded<br>SNPs confirmed by PCR product Sanger sequencing
8	Roetzer A, 2013	HE663067 (EMBL)	454 pyrosequencing (for the initial complete genome sequencing of strain 7199/99); Illumina platform (for resequencing of all isolates)	Average coverage: 96.4%	-	Assembly using GS De Novo Assembler (for 454 reads); Illumina reads mapped to H37Rv reference using SARUMAN exact alignment program; SNPs extracted with customized Perl scripts	H37Rv (NC_000962)	Minimum coverage: 10 reads; Minimum allele frequency: 80% for SNP detection; SNPs in repetitive regions (PPE, PE_PGRS, ESX genes) were manually validated or excluded to avoid false positives
9	Walker TM, 2013	Please see Supplemetary table 2	Illumina HiSeq platform	Average coverage: 88.5%	-	Mapping: Stampy v1.0.13 (without BWA premapping); Variant calling: SAMtools mpileup	H37Rv (NC_000962)	Minimum coverage: 5 reads (including one in each direction); Minimum consensus: 75%; Variants excluded if they occurred at sites with unusual depth or within 12 bp of another variant
10	Colangeli R, 2014	No details were available	ABI SOLiD 5500XL platform	Average coverage: 98%	Average depth: 120	Mapping using CLC Genomics Workbench	H37Rv (NC_000962)	Reads trimmed for quality (>20 bases, quality ≥0.05); SNP calls required ≥80% support among reads; repetitive and highly duplicated regions excluded (PE/PPE, PE-PGRS genes); minimum coverage: 10×
11	Eldholm V, 2014	PRJEB5899 (BioProject identifiers)	Illumina HiSeq and MiSeq platforms	-	Median depth:	Reads were aligned to the M.	H37Rv	Thresholds were SNP ≥95% frequency, depth ≥20, Q-score 60.

					210	tuberculosis H37Rv genome using SeqMan NGen (DNASTAR). SNPs were called in SeqMan Pro and a haploid Bayesian approach in SeqMan Ngen		Repetitive regions, PE/PPE genes, and nearby SNPs (<10bp apart) were excluded
12	Cohen KA, 2015	PRJNA183624 and PRJNA235615 (BioProject identifiers)	Illumina HiSeq 2000 platform	Median coverage: 99.9%	Median depth:143	Reads were mapped using BWA v0.5.9. For SNP identification, Pilon v1.5 was used	H37Rv (CP003248.2)	-
13	Guerra-Assunção JA, 2015	PRJEB2794 and PRJEB2358 (BioProject identifiers)	Illumina HiSeq 2000 platform	-	-	Mapping using BWA-mem; SNP calling with SAMtools	H37Rv (NC_000962)	Minimum read length 50 bp, nucleotides >Q27, reads with >15% missing genotypes excluded; minimum SNP calling criteria: 20× depth, majority allele ≥75% frequency; repetitive regions (PE/PPE genes) excluded
14	Copin R, 2016	PRJNA312221 (BioProject identifiers)	Illumina platform	-	-	Reads aligned using BWA; variant calling with SAMtools and ANNOVAR for annotation	H37Rv (NC_000962)	Polymorphic sites were considered valid if minor allele frequency >10%, coverage ≥50 reads forward and reverse, base quality >20, mapping quality >19, no indels, minimal strand bias (p < 0.01 Fisher's exact test); PE/PPE and phage regions excluded
15	Korhonen V, 2016	No details were available	Illumina MiSeq platform	-	-	-	-	SNPs supported by at least two reads on each strand with >70% of mapped reads, mapping quality ≥45; repeat regions (~9.3% of genome) excluded from analysis
16	Lillebaek T, 2016	PRJEB10245 (BioProject identifiers)	Illumina MiSeq and HiSeq2000 platforms	-	-	BWA v0.7.10 for read mapping; GATK for realignment around indels; SAMtools v0.1.19 (mpileup and bcftools) for raw variant calling; scaffolds assembled with SPAdes; SNP validation by visual inspection in Geneious	H37Rv (NC_000962), or Mtb CTRI-2 (CP002992)	Minimum mapping quality (mapQ) = 30 for raw variant calling<br>SNPs supported by ≥5 reads and at least one read in each direction<br>Minimum average mapQ of SNPs = 45<br>For H37Rv: only homozygous calls (0/0 or 1/1) used<br>For CTRI-2: variants supported by ≥85% of reads were considered fixed

17	Folkvardsen DB, 2017	PRJEB20214 (BioProject identifiers)	Illumina MiSeq and NextSeq 500 platforms	-	-	Reads mapped with BWA.<br>Variant calling with SAMtools and bcftools	Mtb H37Rv reference genome	Minimum read depth of 10.<br>Minimum variant coverage of 5 reads.<br>SNPs considered if frequency >85%.<br>SNPs in repetitive regions (PE, PPE, PE_PGRS genes, and transposons) were removed
18	Herranz M, 2018	ERS2016357-ERS2016427 and ERP002297 (http://www.ebi.ac.uk)	Illumina MiSeq	-	Average depth: 87	Alignment with BWA and confirmation with MAQ<br>Variant calling using Samtools	-	Minimum coverage >20x<br>Mapping quality ≥20<br>Homozygous SNPs: ≥90% of reads<br>Heterozygous SNPs considered only if observed in homozygous state in other isolates<br>Variants in repetitive/phage/PE-PPE regions, near indels, or in SNP hotspot regions (>3 SNPs within 10 bp) were excluded
19	Kühnert D, 2018	PRJEB5925 (BioProject identifiers)	Illumina HiSeq 2000, and Illumina Genome Analyzer Iix	-	-	SNPs determined with SAMtools; reads mapped using Burrows-Wheeler Aligner (BWA) and SMALT;	-	Minimum coverage of 10 reads; minimum phred-scaled mapping quality of 20; SNPs in PE/PPE/PGRS, maturase, phage, insertion sequence, or 13E12 repeat family protein genes removed
20	Merker M, 2018	Please see Supplementary file 1 (correspoding artcile)	Illumina Technology (MiSeq and HiSeq 2500)	-	-	Mapping with BWA; alignments refined with GATK and SAMtools	H37Rv (NC_000962)	Variants called if: minimum of 4 reads in both forward and reverse orientation, 4 reads supporting the allele with phred score ≥20, and ≥75% allele frequency. Repetitive elements and Indels excluded from phylogenetic reconstructions.
21	Xu Y, 2018	SRR3742653-SRR3742670 (GenBank)	PacBio Single-molecule real-time sequencing	Coverage: 99%	Depth: 138 to 270	Alignment: SOAP2 for mapping reads to reference genomes.<br>SNP Calling: SOAPsnp.	H37Rv (NC_000962)	Minimum read coverage at SNP site > 3.<br>Illumina quality score for each allele > 30.<br>Number of mapped best bases > 2× number of second-best mapped bases.<br>SNPs located in PE/PPE and PE-PGRS families were excluded.
22	Colangeli R, 2020	PRJNA607763 (BioProject identifiers)	Illumina HiSeq 2500	Average coverage:97.3%	Average depth: 498	Alignment using Bowtie 2 (v2.2.6);<br>SNP calling using SAMtools (v1.2)	H37Rv (NC_000962)	At least 75% read support<br>Minimum of 4 reads in forward and reverse directions

						and BCFtools (v1.2)		Minimum Phred score of 20<br>Exclusion of SNPs in repetitive regions (PE/PPE genes) and drug-resistance genes<br>Minimum coverage of 20 reads for SNPs (in SNPTB pipeline)
23	Godfroid M, 2020	Please see Supporting information: S1 Table. Samples and accession numbers	-	-	-	seven variant callers (GATK, FreeBayes, Delly, Pindel, SvABA, Scalpel, and MindTheGap) were applied; re-genotyping (GATK for SNPs and short indels, svtyper for long deletions) and back-genotyping were performed to validate variants.	MTB strain 49–02 (RefSeq: NZ_HG813240.1)	Variants retained with allele frequency >75%.<br>SNPs were excluded if undetermined in >5 samples; indels excluded if undetermined in >20 samples.
24	Bainomugisa A, 2021	PRJNA639216 (BioProject identifiers)	Illumina HiSeq 2000 and 4000	-	-	Read mapping: BWA (Burrows-Wheeler Alignment)<br>Alignment refinement: SAMtools and GATK<br>SNP and indel calling: GATK UnifiedGenotyper	H37Rv (NC_000962)	Variants required: minimum read depth of 10 (≥5 reads in each forward and reverse direction)<br>Phred score >30<br>Strand bias <0.6<br>Allele frequency >75%<br>Variants in repetitive regions (like PE/PPE genes) or within 10 bp of indels were excluded
25	Comín J, 2022	SAMN26037035-SAMN26037070 (GenBank); PRJNA808219 (BioProject identifiers)	Ion Torrent technology	Coverage: 97.4%	-	Read mapping: Bionumerics and additional validation using Snippy and IGV<br>SNP annotation: Snippy software and manual confirmation with Integrative Genomics Viewer (IGV)	H37Rv (NC_000962)	-
26	Sadovska D, 2023	PRJEB53131 (BioProject identifiers)	Ion Proton system	-	Median depth: 57.4	Tools: Filter by Quality v1.0.2, Trim Galore! v0.6.3, Filter FASTQ v1.1.5	H37Rv (NC_000962)	Quality control: Phred ≥10 for 95% of nucleotides and ≥20 for 80% of nucleotides

						SNV calling and mapping: Snippy v3.6.0		Adapter trimming and low-quality trimming (Phred <20)<br>Reads >200 bp discarded<br>Variant calling: Minimum 4 reads, both forward and reverse strand support, mapping quality >20, allele frequency ≥90%<br>Variants in repetitive regions, PE/PPE genes, or near indels (5 bp window) discarded
27	Zhang G, 2024	No details were available	Illumina MiSeq platform	-	-	Read alignment: BWA<br>Variant calling: bcftools	H37Rv (NC_000962)	Mapping quality ≥30<br>Base quality ≥20<br>≥20 mapped reads on each strand<br>Variant quality score (QUAL) ≥60<br>Excluded: reads unmapped, duplicate, or low quality; variants in PE/PPE and mobile genetic element regions
